# Vascular hypoperfusion in acute optic neuritis is a potentially new neurovascular model for demyelinating diseases

**DOI:** 10.1371/journal.pone.0184927

**Published:** 2017-09-19

**Authors:** Ta-Ching Chen, Chao-Yuan Yeh, Chao-Wen Lin, Chung-May Yang, Chang-Hao Yang, I-Hung Lin, Pao-Yang Chen, Jung-Yu Cheng, Fung-Rong Hu

**Affiliations:** 1 Department of Ophthalmology, College of Medicine, National Taiwan University, Taipei, Taiwan; 2 Graduate Institute of Clinical Medicine, College of Medicine, National Taiwan University, Taipei, Taiwan; 3 Department of Pathology, University of Southern California, Los Angeles, California, United States of America; 4 School of Medicine, College of Medicine, National Taiwan University, Taipei, Taiwan; 5 Institute of Plant and Microbial Biology, Academia Sinica, Taipei, Taiwan; 6 Department of Healthcare Information and Management, Ming Chuan University, Taoyuan, Taiwan; Bascom Palmer Eye Institute, UNITED STATES

## Abstract

**Purpose:**

Optic neuritis is highly correlated with multiple sclerosis and is a major cause of acute visual loss and long-term neuronal degeneration. Primary cerebral hypoperfusion has been reported in brain demyelinating diseases. This study investigated whether peripapillary perfusion is changed in patients with acute optic neuritis (AON).

**Methods:**

This three-year cohort study was conducted from September 1 2012, to August 31, 2015. Two hundred and forty-one patients with non-glaucomatous acute optic neuropathy were screened, and 42 non-highly myopic patients who had suffered their first episode of unilaterally idiopathic AON were studied. All cases received spectral-domain optical coherence tomography (OCT) examination, general survey, and standard corticosteroid therapy. OCT images were analyzed using a customized MATLAB program for measuring peripapillary choroidal thickness (PCT). Multivariate regression models were constructed to identify factors that are significantly related to peripapillary perfusion.

**Results:**

Decreased PCT was found in eyes experiencing AON combined with disc swelling (the ratio of lesion eye PCT/fellow eye PCT was 0.87 ± 0.08; range, from 0.75 to 1.00). In comparison to the healthy fellow eyes, approximately every 26% increase in the thickness of the retinal nerve fiber layer due to axonal swelling was associated with a 10% decreased thickness of PCT. Thinner PCT is also correlated with poorer trough vision, which may lead to poorer final vision. These findings were obvious in patients with optic papillitis but not in patients with retrobulbar neuritis.

**Conclusions:**

Peripapillary vascular hypoperfusion was found in patients experiencing AON combined with disc swelling. These findings are unlike those for other ocular inflammatory diseases but are consistent with cerebral hypoperfusion, which is found in brain demyelinating diseases; thus, these findings may represent a new neurovascular model in this field.

## Introduction

Optic neuritis (ON) is usually a primary demyelinating process and is a major cause of acute visual loss in young to middle-aged adults. Isolated acute optic neuritis is among the most common first manifestations of multiple sclerosis (MS) and neuromyelitis optica (NMO), which are leading causes of neurologic disability with important socio-economic impacts.[1–3] Following episodes of acute ON, patients often suffer irreversible functional and structural damage to the optic nerve. In 2000, the Optic Neuritis Treatment Trial (ONTT) reported that after 5–8 years of follow-up, eyes affected by ON were prone to decreased contrast sensitivity, poorer visual acuity, impaired color vision, and visual field defects.[4] In the past decade, with advances in imaging techniques including optical coherence tomography (OCT), the structural changes that occur during ON can be studied more easily. It is widely accepted that the retinal nerve fiber layer (RNFL) atrophies after episodes of ON, which is representative of optic nerve axon degeneration.[5–9] Furthermore, recent evidence has shown that outer retinal neurons might also be involved.[10]

Despite the ability of the current standard treatment (high-dose intravenous corticosteroid) to speed the resolution of inflammation, the treatment does not seem to influence visual outcome or atrophy of the optic nerve.[1] Scientists continue to experience difficulty in finding effective treatments that provide neuroprotection to prevent or reverse long-term visual dysfunction.[11–15] Similar to the neurodegenerative process of multiple sclerosis, optic atrophy resulting from ON is believed to result from the immune-mediated inflammatory demyelination of optic nerve axons, leading to axonal injury and secondary apoptosis of retinal ganglion cells.[16–17] However, in MS, neurodegeneration can continue to progress despite profound immunosuppression.[18–19] As recently highlighted by delicate perfusion-weighted magnetic resonance imaging (MRI) techniques, decreased cerebral perfusion in patients with MS has attracted great interest.[20–24] Cerebral hypoperfusion can be observed in different stages and subtypes of patients and might be regarded as part of MS pathophysiology.[25–28]

Currently, ON represents a promising target for trials of neuroprotective therapies of multiple sclerosis.[11–15, 29] Based on the concepts outlined above, it is important to determine whether vascular hypoperfusion also plays a role in the neurodegenerative process in patients with acute ON. However, it is difficult to quantify this blood perfusion precisely using traditional imaging techniques. Recent advances in spectral-domain OCT with enhanced depth imaging have revolutionized the field by enabling the detailed evaluation of choroidal thickness.[30] Furthermore, several studies have shown that choroidal thickness directly or indirectly reflects choroidal circulation.[31–33] The choroid supplies blood to retinal pigmented epithelium (RPE), photoreceptors, and the prelaminar portion of the optic nerve.[34–35] Choroidal thickness in the macular area has been proven to be related to a variety of ocular pathologies, such as diabetic retinopathy, high myopia, age-related macular degeneration, and central serous chorioretinopathy.[36–39] The evaluation of peripapillary choroidal thickness (PCT) is further applied to optic nerve diseases and disorders that are related to the peripapillary area, including nonarteritic anterior optic neuropathy (NAION), glaucoma, high myopia, and pseudoexfoliation syndrome.[40–45] In this study, we analyzed the association between vascular perfusion and acute optic neuritis within a complete 3-year institutional cohort of patients with idiopathic acute optic neuritis in National Taiwan University Hospital (NTUH). These data were further correlated to their baseline profiles and neurological progression. To the best of our knowledge, this is the first systemic cohort study on this topic.

## Materials and methods

### Study population

**Fig 1:**
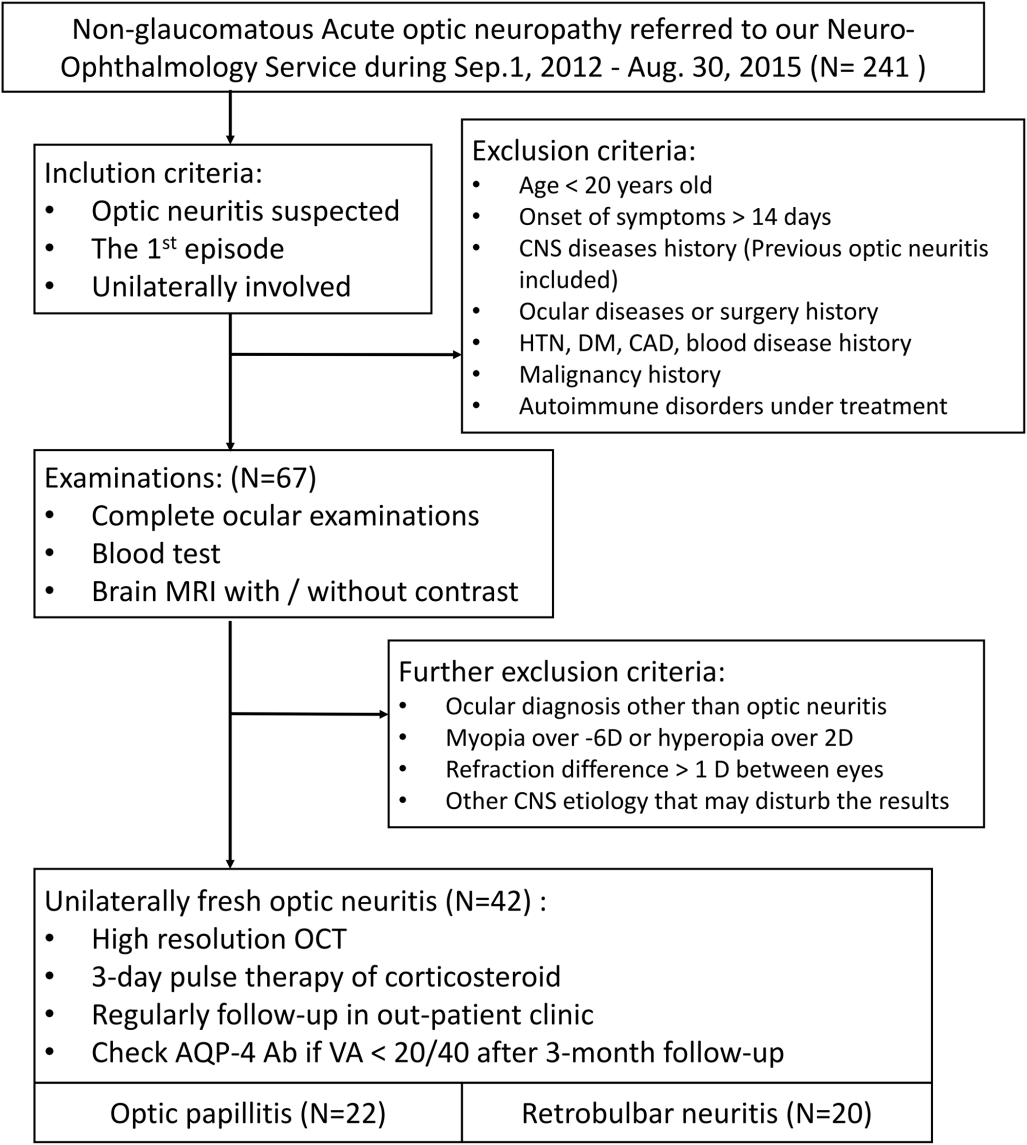
Flow chart of the inclusion and exclusion criteria applied in this study.

Patients at the National Taiwan University Hospital who experienced acutely unilateral optic neuritis for the first time between September 1^st^, 2012 and August 31^st^, 2015 were selected for inclusion in this institutional cohort study. Patients suffering from non-glaucomatous acute optic neuropathy (N = 241) who had been referred to our neuro-ophthalmology service were initially included. After excluding patients who did not match the inclusion criteria and/or had a history matching the exclusion criteria (please see Fig 1 for details), 67 patients were enrolled. These criteria were chosen to avoid diseases that might interfere with the evaluation of RNFL thickness and choroid hemodynamics. After serial examinations, a further 25 cases were excluded because of a non-ON diagnosis or refractive errors that might affect choroidal thickness. Overall, a total of 42 patients who had completed the examinations and treatments were enrolled in this study. Written informed consent forms were obtained from the study participants. The study conformed to the Tenets of the Declaration of Helsinki and was approved by the Research Ethics Committee of National Taiwan University Hospital (NTUH201409073RIND).

### Medical history

As the patients were referred to our service, their medical records were reviewed, and data was collected regarding age, gender, and past diagnosis and/or treatment of (1) central nervous system disorders including MS and NMO, (2) malignancy, (3) ophthalmological surgery or diseases, (4) autoimmune disorders, or (5) chronic diseases such as diabetes mellitus (DM), hypertension (HTN), cardiac artery disease (CAD), and blood diseases. These data were doubly confirmed by (1) recording the medical history from patients and their family and (2) examining the ICD-9 diagnosis record at Taiwan’s National Health Insurance system. We excluded patients having any of the diseases listed above to eliminate possible interference with measurements of choroidal thickness.

### Ocular examinations

All participants were given complete ophthalmological examinations including pupillary light reflex, best-corrected visual acuity (BCVA), Ishihara color vision test, cycloplegic refraction, intraocular pressure (IOP), and indirect fundus examination. Visual field (VF) test was examined using the central 30–2 program (Humphrey 740i Visual Field Analyzer, Carl Zeiss Meditec, Dublin, US). Pattern-reversal visual-evoked potential (VEP) test was carried out according to the standards of the International Society for Clinical Electrophysiology of Vision (ISCEV) (UTAS Visual Testing System with SunBurst^™^ Ganzfeld, LKC Technologies, Gaithersburg, US).[46] Cases who had (1) a spherical equivalent of greater than +2 diopters (D) in hyperopia, (2) greater than -6 D in myopia, or (3) anisometropia of greater than 1 D between eyes were excluded. A Landolt-C optotype was used to measure visual acuity. Visual acuity was then converted to the logarithm of the minimum angle of resolution (LogMAR) for statistical analysis. Based on the initial presentation of optic disc appearance through fundus examination and photography, these 42 participants were further divided into two subgroups: an optic papillitis (OP) group presenting a visibly swollen disc demonstrating acute papillitis and a retrobulbar neuritis (RN) group presenting no signs of disc swelling. Quantified data regarding the segmental and average thickness of the retinal nerve fiber layer (RNFL) was obtained using spectral-domain optical coherence tomography (OCT) imaging, as described below. All patients assigned to the OP group showed at least 10% thicker average RNFL compared to their healthy fellow eyes, and none of the patients in the RN group demonstrated this trend.

### Brain imaging and systemic evaluation

Blood samples were obtained at the time that participants were referred to our service and before they received pulse corticosteroid therapy. We collected data regarding complete blood cell count, differential count, electrolytes (serum sodium and potassium concentrations), liver enzymes (aspartate transaminase (AST) and alanine transaminase (ALT)) and renal function indicators (blood urea nitrogen (BUN) and serum creatinine (Cr)), autoimmune profiles including antinuclear antibody (ANA), rheumatoid factor (RF), complement components C3 and C4, a venereal disease research laboratory test (VDRL), C-reactive protein (CRP), and the erythrocyte sedimentation rate (ESR) for 1 and 2 hours. These tests were used to determine baseline health status. If there were abnormal data indicating specific diseases, we performed a more in-depth survey and referred the patient to relevant specialists. We excluded cases with renal insufficiency, hepatitis, blood diseases, active infections, and ocular inflammation due to syphilis. Positive findings regarding autoimmune profiles that were found in this examination were not used to exclude patients, but these individuals were simultaneously referred to a rheumatologist for further evaluation and follow-up. Aquaporin-4 antibody was tested if the patient did not achieve a visual acuity of better than 20/40 within 3 months. Brain MRI scans with and without contrast were acquired using a Siemens Sonata 1.5-T scanner. The results were read by an experienced radiologist, and the lesion numbers, locations and sizes were recorded within a formal medical report. Cases with brain pathology that might influence the visual pathway were also excluded.

### Optical coherence tomography (OCT) imaging with MATLAB analysis

Patients underwent disc and RNFL imaging with spectral-domain OCT (SD-OCT, RTVue RT-100, Optovue Inc. Fremont, CA, USA). The segmental and average thickness of RNFL were calculated in an automated manner, and the results were presented in an ONH (optic nerve head) analysis report. In addition to RNFL imaging, the high-resolution crossline imaging mode of the RTVue was used to obtain a high-resolution cross-sectional image of the peripapillary retina and choroid along an 8-mm line passing through the central part of the disc. Two sets of images for each disc (X-Y axis (0 and 90 degree) and oblique axis (45 and 135 degree)) were recorded to obtain peripapillary images in 8 directions. The outer border of the choroid can be well visualized on the single-line image for most patients. Images with signal strength ≥7 were included in the study.

**Fig 2:**
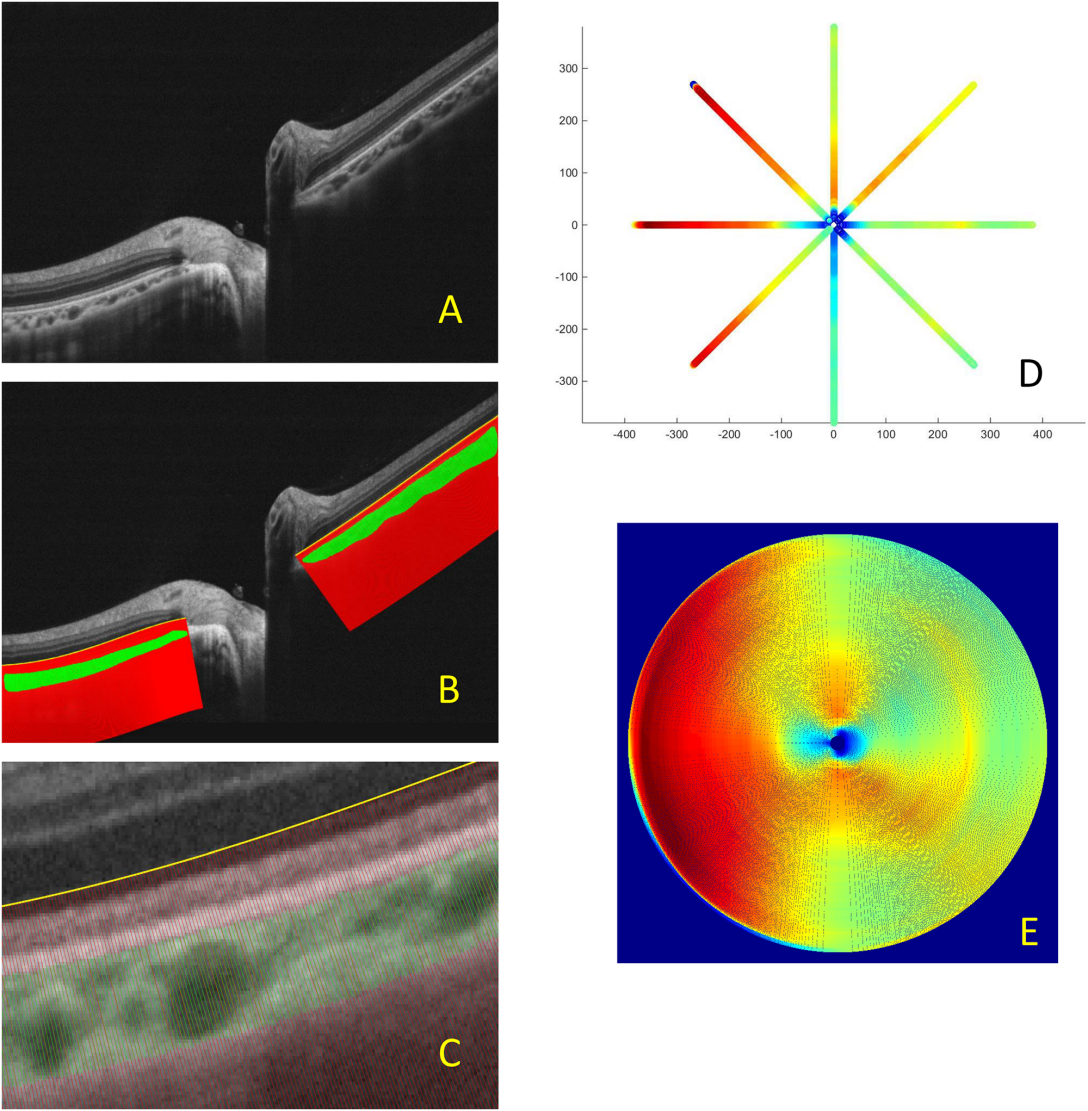
Computer-assisted analysis of peripapillary choroidal thickness (PCT). (A)High-resolution OCT image of the peripapillary retina and choroid along an 8-mm line passing through the central part of the disc.(B)The yellow line represents the reference curve, and auxiliary red lines perpendicular to the yellow curve were drawn at a fixed 1-μm interval for calculating the choroid thickness at high spatial density.(C)A close-up of (B), showing auxiliary lines.(D)An illustration of the calculation of choroid thickness based on 4 cross-line images that cover eight regions of the eye. Warm colors represent thicker choroid regions while cool colors represent thinner choroid regions.(E)A full circle of PCT derived from a spline interpolation of the data shown in (D).

No commercial software is available for the automated measurement of peripapillary choroidal thickness (PCT). Furthermore, more than half of the acute ON eyes (22 of 42) had a swollen disc, which complicated the evaluation of PCT. To unleash the true diagnostic power of OCT, we developed a custom-written MATLAB script for the semi-automated measurement of PCT at high spatial resolution and to visualize whole-field peripapillary choroid thickness using data interpolated from a limited number of cross-line scans. To obtain an original image that would provide the best image-processing results, we first implemented a file reader to extract raw image data from the OCT files generated by RTVue (Fig 2A). All following measurements were completed by two masked reviewers (IH Lin and HM Hsu). The choroid boundary was manually marked using a free-hand region of interest tool. A reference curve was then manually drawn parallel to Bruch’s membrane to allow choroid thickness measurement truly vertical to the retina (Fig 2B and 2C, both green areas show the choroid). To remove artifacts caused by trembling hand motion while drawing the curve that may lead to erroneous calculation, the curve was fit to a polynomial function to generate a smoothed curve (Fig 2B and 2C, yellow line). Auxiliary lines perpendicular to the retinal curvature were drawn at a fixed interval to calculate choroid thickness at a high spatial density (~1 μm intervals) (Fig 2B and 2C, red lines). For OCT images centered on the optic disc, the center of the image was determined by averaging the innermost coordinates of the two reference curves, which were presented as the termination of Bruch’s membrane. Disc diameters were compared between the left and right eyes in the same participant.

Choroid thickness was calculated from 4 cross-line images covering eight regions of the eye (0°, 45°, 90°, 135°, 180°, 225°, 270°, and 315°) (Fig 2D). In all 42 cases, the lengths obtained lay in the range from 2.83 to 3.18 mm. To aid in visualizing the choroidal topography and statistical analysis, data for eight regions were trimmed down to the same size (2.80 mm in diameter), and spline interpolation was used to fill the data points in the full circle (Fig 2E). In every picture, to eliminate inaccurate evaluation of the edge, we further trimmed the diameter to 2.50 mm for statistical analysis of the segmental and average thickness. Whole average thickness (reported as PCT) was defined as the average over the circular area. Temporal half average thickness (reported as TemPCT) was defined as the average over the temporal 180-degree semicircular area. Every OCT picture was evaluated three times by each observer at a consecutive one-week interval. The choroidal thickness analyzed represents the average of three repeats from 2 reviewers. Differences in average thickness of more than 10% between the two independent observers were considered inaccurate, and the picture was further evaluated by a third reviewer (CY Yeh). The final estimate was obtained as the average of the two closer measurements. The MATLAB script (S1 File) for the measurement of choroidal thickness has been deposited in the website listed below: https://www.protocols.io/private/553f318d0d6746b96df18b8b448ea365.

### Statistical analysis

A database was established to facilitate data management and statistical analysis. Subsequent statistical analyses were carried out using SPSS 12.0 (SPSS Inc., Chicago, IL, USA) and SAS 9.1.3 (SAS Institute Inc., Cary, NC, USA) software. Two-sided P-values ≤ 0.05 were considered statistically significant. Two-sample t tests were used to compare the mean values of continuous variables between the optic papillitis and retrobulbar neuritis groups. One-way ANOVA test was used for comparisons among more than two groups. Chi-square tests or Fisher’s exact tests were used to examine the associations between categorical variables listed in Tables 1 and 2. We constructed multivariate regression models to examine the variation of PCT and visual impairment. The goals were to identify predictive factors and estimate their effects. Univariate regression models were first used to select individual factors that are predictive. These selected factors were later included in the multivariate models. In the final models, we report the factors that are significant (p≤0.05) and their effect estimates. The analyses were performed on the following three categories: (1) all 42 samples, (2) the optic papillitis group, and (3) the retrobulbar neuritis group.

**Table 1:** Baseline characteristics of the study population.

		Optic Papillitis	Retrobulbar Neuritis	P-value
Number of cases		22	20	
Mean age (age range, years)		37.1 ± 12.2 (20–57)	39.1 ± 11.8 (21–58)	0.58
Gender (M / F)		7 / 15	5 / 15	0.63
Lesion eye (OD / OS)		12 / 10	10 / 10	0.78
Duration to treatment (days)		6.4 ± 2.9	5.8 ± 3.5	0.53
Clinical characteristics	Impaired color vision	100%	100%	
	Painful ocular movement (n)	95% (21)	90% (18)	0.51
	Prolonged P100 latency in VEP	100%	100%	
	VF defect (dB)	-20.4 ± 8.4	-18.0 ± 9.6	0.43
	Nerve enhancement in MRI (n)	68% (15)	60% (12)	0.35
	Brain abnormality in MRI (n)	18% (4)	20% (4)	0.61
	Abnormal autoimmune (+ AQP4) profile (n)	36% (8)	20% (4)	0.02*
Initial BCVA	Better than 20/40	2	4	0.19
	20/400 to 20/40	12	8	-
	Worse than 20/400	8	8	-

M = male; F = female; OD = right eye; OS = left eye; n = number of cases; AQP4 = aquaporin-4; BCVA = best corrected visual acuity;

Two-sample t tests for continuous variables; Fisher’s exact tests for categorical variables.

* indicates P<0.05;

**Table 2:** Compared characteristics between the lesion and fellow eyes and between the two groups.

	Optic Papillitis (n = 22)		Retrobulbar Neuritis (n = 20)		P-value
	Lesion eye	Fellow eye	Lesion eye	Fellow eye	
Refraction (SE, D) [range]	-2.07 ± 2.38 [+1.50 ~ -5.75]	-1.99 ± 2.21 [+2.00 ~ -5.75]	-3.34 ± 2.16 [+1.75 ~ -5.5]	-3.22 ± 2.27 [+2.00 ~ -5.75]	0.10
IOP—initial	15.3 ± 3.3	15.4 ± 3.2	15.1 ± 2.8	15.1 ± 2.6	0.96
IOP– 2 month	15.0 ± 2.0	14.6 ± 2.9	15.5 ± 2.4	15.9 ± 2.7	0.48
Average PCT (μm)	196.9 ± 50.6^1^	211.7 ± 58.2^1^	212.6 ± 33.7	202.5 ± 38.4	
Ratio (L/F) [range]	0.87 ± 0.08 [0.75–1.00]		1.06 ± 0.10[0.90–1.23]		0.19
Temporal PCT (μm)	195.4 ± 56.4^2^	232.4 ± 60.9^2^	216.5 ± 36.6	208.6 ± 51.3	
Ratio (L/F) [range]	0.85 ± 0.16 [0.58–1.27]		1.07 ± 0.20[0.79–1.48]		0.38
Average RNFL (μm)	147.7 ± 24.7^3^	108.5 ± 8.0^3^	103.3 ± 8.5	110.3 ± 9.0	
Ratio (L/F) [range]	1.36 ± 0.18 [1.09–1.71]		0.94 ± 0.04[0.81–0.98]		<0.01
Temporal RNFL (μm)	157.1 ± 25.9^4^	118.0 ± 11.7^4^	117.8 ± 21.6	124.3 ± 18.2	
Ratio (L/F) [range]	1.33 ± 0.19 [1.06–1.76]		0.95 ± 0.07[0.76–1.03]		<0.01

1: P = 0.26, 2: P = 0.48, 3: P<0.001, 4: P<0.001; SE = spherical equivalent; L/F = lesion eye/fellow eye;

2: One-way ANOVA was used in the comparisons among four groups (lesion eye in OP, fellow eye in OP, lesion eye in RN, and fellow eye in RN) in the variables of refraction, IOP-initial, and IOP-2 month; two-sample t test was used in the comparisons between two groups (ratio of PCT and RNFL) in the variables of average PCT, temporal PCT, average RNFL, and temporal RNFL.

### Results

The study included 42 patients with idiopathic acute optic neuritis with unilateral involvement. The average age at referral was 38.0 ± 11.9 years (range from 20–58 years old), and there were more female than male patients (F:M = 30:12). All recruited patients demonstrated positive findings of relative afferent pupil defect (RAPD) signs, decreased central vision, impaired color vision, and newly onset visual field defect. In their diseased eye (lesion eye, LE), disc swelling was observed in 22 patients (the optic papillitis (OP) group) but not in the other 20 patients (the retrobulbar neuritis (RN) group). Table 1 compares the baseline characteristics between the two groups. No significant difference was found in age distribution, gender, left/right eye, duration from symptom onset to medical survey, and initial visual acuity between the two groups. Regarding the diagnostic findings for optic neuritis, 100% of these patients demonstrated impaired color vision and prolonged P100 latency in the VEP test. The two groups also exhibited similar clinical findings, such as painful eye movement, severity of VF defect, optic nerve enhancement and white matter abnormality as seen in a brain MRI study. The only statistically significant difference we found was for positive autoimmune profile (36% in the OP group vs. 20% in the RN group, P = 0.016, Aquaporin 4 antibody test included).

Table 2 lists the refractive status, IOP at the acute stage and 1-month follow-up, and PCT for intra-group (lesion eye vs. fellow eye) and inter-group (OP group vs. RN group) comparisons. The distribution of refraction was not significantly different among the four sub-groups. IOP remained stable during the acute stage of both types of optic neuritis. These observations were made to ensure the reliability of our interpretation of PCT. To eliminate the effect of individual variance in choroidal thickness, we calculated the L/F ratio representing the ratio of (lesion eye) / (fellow eye) in the PCT of every recruited case. In the papillitis group, PCT tended to be thinner in the lesion eyes than in the fellow eyes, in terms of the overall average and TemPCT (L/F ratios of 0.87 ± 0.08 and 0.85 ± 0.16, respectively). Interestingly, none of the 22 cases had a ratio over 1 regarding average PCT (the values ranged from 0.75 to 1.00). However, the difference in thickness between the lesion and fellow eyes did not reach statistical significance (P = 0.26 for average PCT, and P = 0.48 for macular PCT). This lack of significance might be due to the low number of patients. This tendency was not found in patients with retrobulbar neuritis (L/F ratio 1.06 ± 0.10 for average PCT and 1.07 ± 0.20 for TemPCT). The RNFL thickness was also compared between the lesion and fellow eyes based on the L/F ratio. Reasonably, the L/F ratio in the OP group was greater than 1 (average RNFL, 1.36 ± 0.18; macular RNFL, 1.33 ± 0.19) and differed significantly (P<0.001) with that of the RN group (average RNFL, 0.94 ± 0.04; TemRNFL, 0.95 ± 0.07) (P<0.001 for both the average RNFL and TemRNFL comparisons).

**Fig 3:**
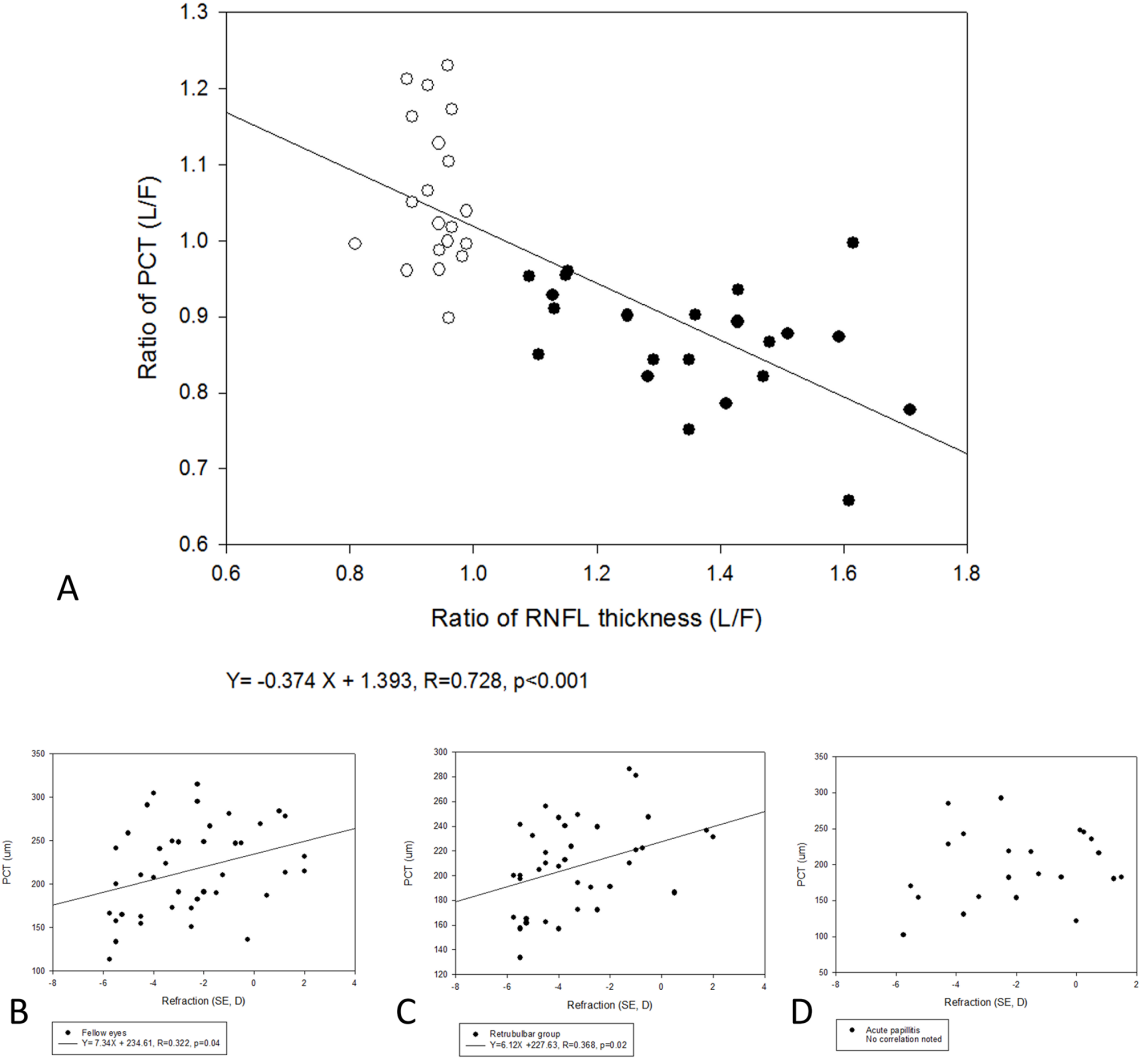
Regression analyses of peripapillary choroidal thickness. (A)The PCT L/F ratio is negatively correlated with the RNFL L/F ratio(B)PCT was weakly correlated with refraction in all fellow eyes.(C)PCT was weakly correlated with refraction in all eyes of patients in the retrobulbar neuritis group.(C)PCT was not correlated with refraction in lesion eyes of patients in the optic papillitis group.(PCT = peripapillary choroidal thickness; L/F = lesion eye/fellow eye; RNFL = retinal nerve fiber layer).

The reverse trends in PCT and RNFL between the OP and RN groups raised the question as to whether peripapillary perfusion is negatively correlated with inflammation of the nearby optic nerve head. Linear regression was conducted to further clarify the relationship between the L/F ratios of PCT and RNFL in our cases (Fig 3A). The black dots in the figure represent patients of the OP group with varying increases of RNFL thickness, and the white dots represent patients of the RN group. The regression equation showed a strong negative correlation; every 26% swelling of the optic nerve head was associated with a 10% decrease of PCT (R = 0.73). Since refractive status has been proven to be a significant factor affecting choroidal thickness in healthy young subjects, we further tested the relationship in (1) all 42 fellow eyes (Fig 3B), (2) both eyes of the RN group (Fig 3C), and (3) eyes that suffered from papillitis (Fig 3D). All fellow eyes and eyes of the RN group both showed low to moderate correlation between PCT and refractive status (R = 0.37 and 0.32, respectively), but no correlation was found for eyes experiencing acute optic papillitis. The results suggested additional factors affect PCT in acute optic papillitis.

Univariable and multivariable regression were used to further clarify the risk factors and predictive parameters associated with the thinning of PCT in eyes with acute optic neuritis. All categorized or quantified data were incorporated in the equation for analysis. In the univariable regression analysis, the presence of disc swelling and the L/F ratio of average RNFL were both strong predictors, while the refractive status of the lesion was also a significant predictor but yielded very small estimates. Multiple regression demonstrated that the best-fit model, when controlled for age and gender, was that which used all three variables, and the RNFL ratio was still the most important variable. Statistical results relating to selected important factors are listed in Table 3. Based on the results of the analysis, the L/F ratio of PCT can be predicted by the following equation:

L/Fratio of PCT = 1.1398 −0.230×L/Fratio of RNFL − 0.0674 (if disc swelling observed)− 0.017×Refraction of the lesion eye (D) + 0.002 ×Age (years) + 0.001 (if male)

The R-square value obtained using this equation was 0.65.

**Table 3:** Regression analysis of the observed variations in peripapillary choroidal thickness.

	Univariate regression		Multivariate regression	
	Estimate	P-value	Estimate	P-value
Age (years)	0.001	0.457	0.002	0.117
Gender	0.004	0.935	0.001	0.985
Lesion eye (OD/OS)	-0.026	0.526		
Duration to treatment (days)	-0.002	0.707		
Disc swelling	-0.191	< .0001^#^	-0.064	0.210
Autoantibodies	-0.066	0.131		
Abnormal brain MRI	-0.021	0.449		
Refraction (D)	-0.020	0.020*	-0.017	0.020*
Refractive difference (L-F, D)	-0.011	0.696		
VF defect (dB)	0.000	0.990		
Average RNFL (L/F ratio)	-0.373	< .0001^#^	-0.230	0.027*
PCT in lesion eye (μm)	0.000	0.369		
			Intercept 1.1398	R-Square 0.6482

* Test of regression coefficients P-value (t-test): P<0.05, # P<0.001; OD = right eye; OS = left eye; D = diopters; L-F = (lesion eye)-(fellow eye); L/F = (lesion eye)/(fellow eye)

Most of our cases experienced good visual recovery after the episode. Thirty-three of the total 42 patients (79%) presented a BCVA of 20/40 or above within three months after steroid treatment. While analyzing the predictors for final vision after this episode, the only parameter that was found significant was “initial trough BCVA” (estimate = 0.144, P = 0.049). Therefore, we used the LogMAR initial trough BCVA as a model to analyze the significant predictors. Statistical results of selected important factors are listed in Table 4. Positive findings in autoimmune profiles and worse VF were correlated with poorer initial trough visual acuity (P = 0.041 and P<0.0001, respectively). The trough visual acuity is defined as the worst BCVA the patient had over some visits close to baseline. The correlation between initial VF defect and trough vision was also significant in the RN subgroup (as found by multivariable regression; P<0.001). However, in the OP group, the L/F ratio for average PCT demonstrated a stronger correlation with initial trough BCVA than any other parameters (estimate = -4.545, P = 0.041). A smaller L/F ratio, representing a thinner PCT in lesion eyes, may lead to more severe visual impairment at the acute stage. However, this phenomenon was not observed in the RN group.

**Table 4:** Regression analysis of initial trough visual acuity (LogMAR).

	All—univariate		All—multivariate		OP group		RN group	
	Estimate	P-value	Estimate	P-value	Estimate	P-value	Estimate	P-value
Disc swelling	0.253	0.314			-	-	-	-
Age (years)	-0.006	0.595	0.001	0.938	-0.007	0.654	-0.003	0.874
Gender	-0.274	0.324	-0.081	0.728	-0.007	0.986	-0.666	0.101
Lesion eye (OD/OS)	-0.033	0.896			-0.187	0.607	0.110	0.763
Autoantibodies	**0.544**	0.041*	0.241	0.306	0.480	0.184	0.556	0.213
Abnormal brain MRI	0.165	0.344			0.448	0.074	-0.117	0.630
Refraction (D)	-0.029	0.602			-0.011	0.898	-0.099	0.247
Duration to treatment	-0.041	0.313			-0.071	0.280	-0.028	0.602
VF defect (dB)	**-0.056**	**< .0001**^#^	**-0.051**	**0.0003**^#^	-0.040	0.060	**-0.068**	**< .0001**^#^
Average RNFL (L/F ratio)	0.490	0.330			0.492	0.624	-5.432	0.205
PCT of lesion eye (μm)	-0.002	0.537			-0.003	0.480	0.002	0.742
Average PCT (L/F ratio)	-0.908	0.354			**-4.545**	0.041*	2.703	0.152
Macular PCT of lesion eye (μm)	0.000	0.893			-0.001	0.800	-0.001	0.815
Macular PCT (L/F ratio)	-0.707	0.236			-1.749	0.114	0.314	0.733
			Intercept	R-Square				
			0.2234	0.3978				

* Test of regression coefficients P-value (t-test): P<0.05, # P<0.001; OD = right eye; OS = left eye; D = diopters; L/F = (lesion eye)/(fellow eye); VF = visual field; RNFL = retinal nerve fiber layer; PCT = peripapillary choroidal thickness; OP = optic papillitis; RN = retrobulbar neuritis

## Discussions

The principal finding of this study is that peripapillary choroidal thickness (PCT) became thinner in eyes with acute optic neuritis and disc swelling (acute papillitis), while no obvious change was seen for retrobulbar neuritis. The phenomenon was positively correlated with disc swelling severity presenting as increased thickness of the retinal nerve fiber layer (RNFL). Simultaneously, lesser PCT is also indicative of poorer trough vision, which may lead to poorer final vision. In both MS and NMO, cerebral hypoperfusion has been mentioned as a pathophysiology. [20–27, 47–48] Some MRI imaging observations have shown that decreased perfusion is correlated with decreased mean diffusivity rather than fractional anisotropy. The finding of ischemia co-existing with acute inflammation is more consistent with primary ischemia than with secondary hypoperfusion after axonal degeneration. [48] Hypoperfusion would worsen mitochondrial energetic failure and oxidative stress, which are gradually recognized as factors that are associated with axonal degeneration. [49–50] Acute optic neuritis is well known to recapitulate several typical characteristics of demyelinating diseases, including inflammatory demyelination, axonal loss, and subsequent endogenous remyelination. [51] Despite the recovery from acute inflammation, such neuronal loss would cause permanent neurological deficit. However, the hemodynamic change in optic neuritis was not systematically evaluated. In our present study, with the aid of high-resolution OCT techniques, we found regional hypoperfusion in patients with acute optic papillitis.

The eye provides a window to intracranial diseases. The development of OCT has facilitated the monitoring of neuronal degeneration in several important diseases, such as Alzheimer's disease, cerebral infarction, occipital injury, and multiple sclerosis. [52–56] New SD-OCT techniques with enhanced depth imaging have further increased the resolution and depth of images, making it possible to evaluate choroidal thickness. The choroid is a vascular structure with several important retinal functions, including the delivery of blood and nutrients, thermoregulation, and the secretion of growth factors. [57] Several diseases have been shown to result in hemodynamic changes based on observations of choroidal thickness under the macular area. [36–39] The macular choroid mainly supplies the outer retina, where the RGC is not located. However, it should be noted that like the peripapillary arterial circle of Zinn-Haller, the peripapillary choroid receives its blood from posterior ciliary arteries. This circle is located in the sclera at the merging point of the optic nerve dura mater with the posterior sclera and supplies blood to the lamina cribrosa of the optic nerve head. [58–59] Therefore, the parameter PCT is a good model of blood perfusion in the anterior optic nerve.

Several factors at baseline can influence an individual's choroidal thickness, including age, refraction, and chronic diseases, such as diabetes. [39, 41, 60–61] Hemodynamics can be further modified by the local environment (for example, uveitis) and local treatment (for example, an intravitreal injection of anti-VEGF agents). [62–65] For this reason, we set very strict criteria to exclude major systemic and ocular diseases that may disturb hemodynamics and excluded patients with high myopia or large anisometropia. Furthermore, to eliminate the effects caused by the compromise of circulation secondary to neuronal degeneration, we excluded all patients with any previous pathology in the retina, optic nerve, and intracranial visual pathway. Finally, we recruited only patients with unilateral optic neuritis and chose the fellow eye as the control to minimize individual variation that may mask the true findings. However, the precise evaluation of PCT remains challenging. The contour is often tilted with high individual variation, and the disc size varies among subjects. Previous studies on PCT have often employed a circle of 3.5-mm diameter on the optic disc to obtain measurements on the circumference. [40–45] To obtain more information and a thorough understanding of the topography of the peripapillary choroid, we designed a MATLAB program to study the choroid through obtaining high-resolution cross-sectional images along 8 directions. The strengths of the approach using our custom program include the following: 1) measurement of the thickness not biased by curvature, 2) high accuracy is obtained as a result of the high measurement density, 3) data interpolation is used for clear visualization and easy point-to-point comparison between the two eyes. The reference plane paralleling Brush’s membrane could eliminate the error resulting from the curved contour. The 2-D data along 8 directions were integrated by spline interpolation to mimic a 3-D topography. Disc diameters were compared between the left and right eyes in every case to eliminate any inaccuracy in judging the disc margin, especially for swollen discs. Using these procedures, we can be more confident of the results obtained.

Our results revealed that PCT is compromised in acute optic neuritis, especially when inflammation is located nearby (i.e., in the papillitis group). Regional hypoperfusion should not be secondary to axonal degeneration since all the recruited cases were free from previous optic nerve and retinal disorders. The result obtained differs from the results expected based on the previous concept that the choroid tends to thicken in ocular inflammatory diseases, such as uveitis and CSCR. [37, 63–65] On the other hand, the result obtained looks similar to the cerebral hypoperfusion that has been reported to occur in MS. Some recent studies found that impairment in functional cerebrovascular pathophysiology may be the underlying cause of neurodegeneration in MS and that the impairment may be mediated by endothelin-1 (ET-1). [27, 66–67] It has also been mentioned that extraocular blood flow can be decreased in patients with MS and that serum ET-1 concentrations are elevated. [68] It is believed that excess ET-1 is secreted by reactive astrocytes. Thus, we propose the hypothesis that in acute optic papillitis, reactive astrocytes around the optic nerve head might release ET-1 to further compromise the surrounding perfusion.

Statistical analysis also showed that choroidal hypoperfusion is positively correlated with the severity of swelling of RNFL, which raises another possibility—that regional hypoperfusion arises due to physical compression from the swelling anterior optic nerve. However, this possibility is less likely since the choroid receives many inputs, including long and short posterior ciliary arteries. Many of these arteries leave the dura mater far from the optic nerve head and are unlikely to be restricted. Second, patients in the RN group, for whom the inflammation was located in a more posterior part of the nerve, showed no real-time change in PCT. Furthermore, a segmental analysis of RNFL, PCT, and the correlation between these was performed in our study, but no significant findings were obtained.

Finding ways to improve neuroprotection in patients with demyelinating diseases has been a major problem. In this respect, due to the good accessibility of visual structures and established vision-related outcome measures, optic neuritis has been a good model, and several clinical trials are ongoing. [11–15, 29] A promising agent undergoing a multi-center trial is erythropoietin (EPO), intravenously injected at 33,000 international units (IU) per day for 3 days in addition to methylprednisolone pulse therapy. [12–13] In the EPO treatment group, significant protection of the RNFL was determined by OCT. The authors declared that EPO might show neurotrophin-like properties in models of ischemia, trauma, epilepsy and MS and might therefore be a modulator in neurodegeneration. [13, 69] In this view of ischemia, EPO activates signaling cascades that increase the brain's resistance to ischemia-reperfusion stress by stabilizing mitochondrial membranes, thus limiting the formation of reactive oxygen and nitrogen intermediates. EPO also suppresses pro-inflammatory cytokine production and neutrophil infiltration. [70] Therefore, if vascular hypoperfusion is part of the pathophysiology in acute optic neuritis, then the functions of EPO in terms of neuroprotection may be greater than those suggested by our imaging results and warrant further exploration.

Our study has several limitations. First, this was a single hospital-based observational study; although this hospital is the most long-standing medical center in Taiwan, some significance values might have been underestimated due to the limited sample size, especially when we set strict criteria for recruitment. Second, the follow-up interval was not long enough to examine the correlations with long-term outcomes, such as the MS conversion rate or retinal degeneration. However, we tried our best to analyze the associations between vascular hypoperfusion and visual outcome. Third, the patient population was purely comprised of Asian individuals; therefore, the results should be carefully interpreted in light of the heterogeneity of different races/ethnicities.

Nevertheless, our study also has several strengths. First, to the best of our knowledge, this is the first published study to have systemically analyzed peripapillary perfusion in acute optic neuritis. Second, we designed a new computerized program to analyze images and to maximize the information that we can obtain through non-invasive OCT imaging techniques. Third, by using strict criteria for recruitment, including disease history and refractive status, the interference of confounding factors in evaluation of PCT should have been minimized.

In conclusion, vascular hypoperfusion in the peripapillary area was found in eyes exhibiting acute optic neuritis and disc swelling. This phenomenon was positively correlated with the severity of nerve swelling and was associated with poorer trough vision, which may lead to poorer final vision after the episode. This finding may correspond to the cerebral hypoperfusion that occurs in brain demyelinating diseases and may represent a new neurovascular model in this field. Hopefully, these results might also lead to new insights for future therapy in the field of neuroprotection.

## Supporting information

S1 File Analysis of choroid thickness on OCT image using MATLAB. (DOCX)
