# Supplementary material for: Vascular hypoperfusion in acute optic neuritis is a potentially new neurovascular model for demyelinating diseases
Source: PLoS One. 2017 Sep 19;12(9):e0184927. doi: 10.1371/journal.pone.0184927 (PMC5605049; doi:10.1371/journal.pone.0184927)

**S1. Analysis of choroid thickness on OCT image using MATLAB**

Steps

- 1 Make sure you have MATLAB 2016b (or newer) with image processing toolbox installed on your computer.
- 2 Dowload the MATLAB scripts from <http://68.181.92.180/~Joe/OCT_image_analysis.zip>
- 3 On the Optovue OCT machine, export cross line OCT images. To allow inperpolation, four cross line images are required for each eye. Single cross line image can still be processed by ***OCT_ImageAnalysis_for_CrossLine.m.***
- 4 Run ***OCT_file_processing.m*** to process OCT images. This script works on raw image generated by Optovue OCT machines. It will make the file name easier to handle and extract image content from cross line scans and line scans and save them into tiff format.
- 5 Run ***OCT_ImageAnalysis_for_CrossLine.m*** through all the tif files.
- 6 Follow the prompt and draw along the contour of the retina on the left side of the image.


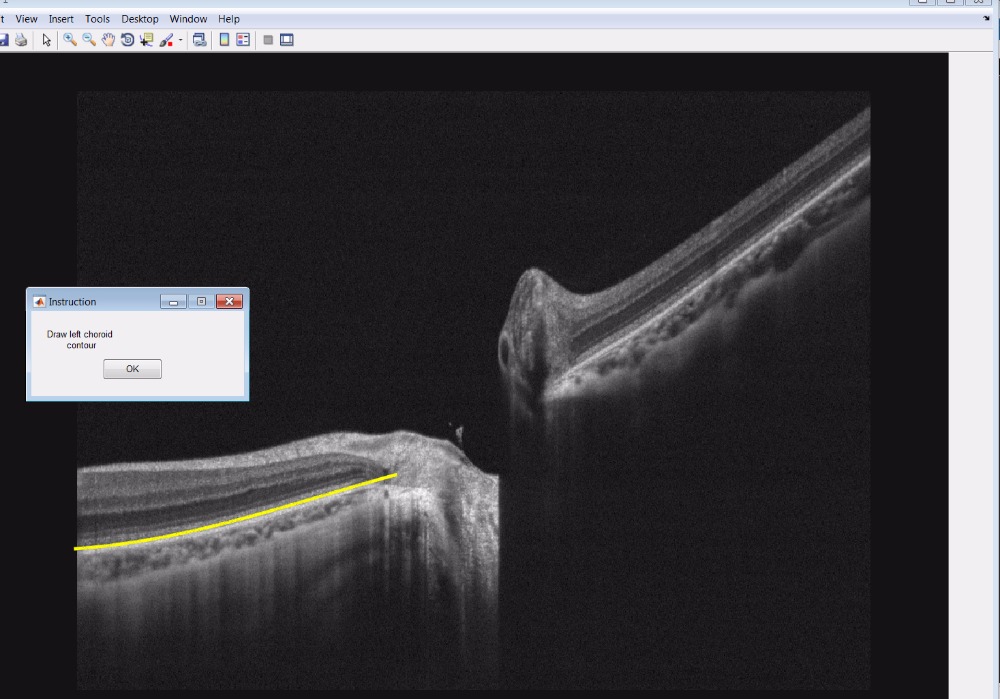


Then follow the prompt and draw along the contour of the choroid on the left side of the image.


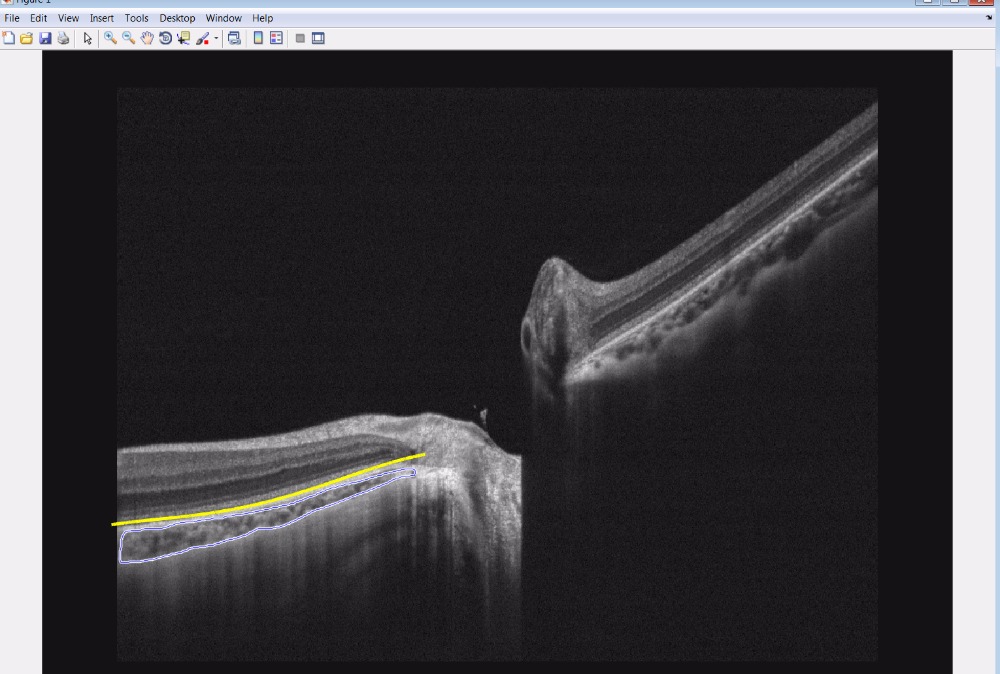


After you're done, choroid thickness will be calculated for you. Red lines represent auxiliary lines perpendicular to the curve. Green lines represent calculated thickness of the curve.


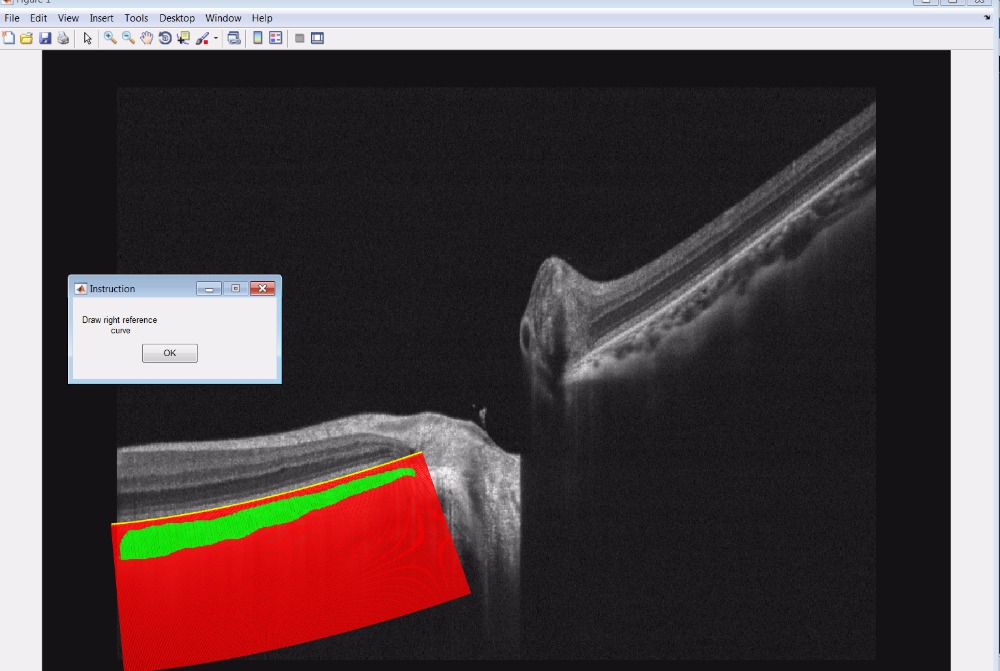


Repeat this process for the right side of the image. Another figure will appear and summarize the thickness measurement.


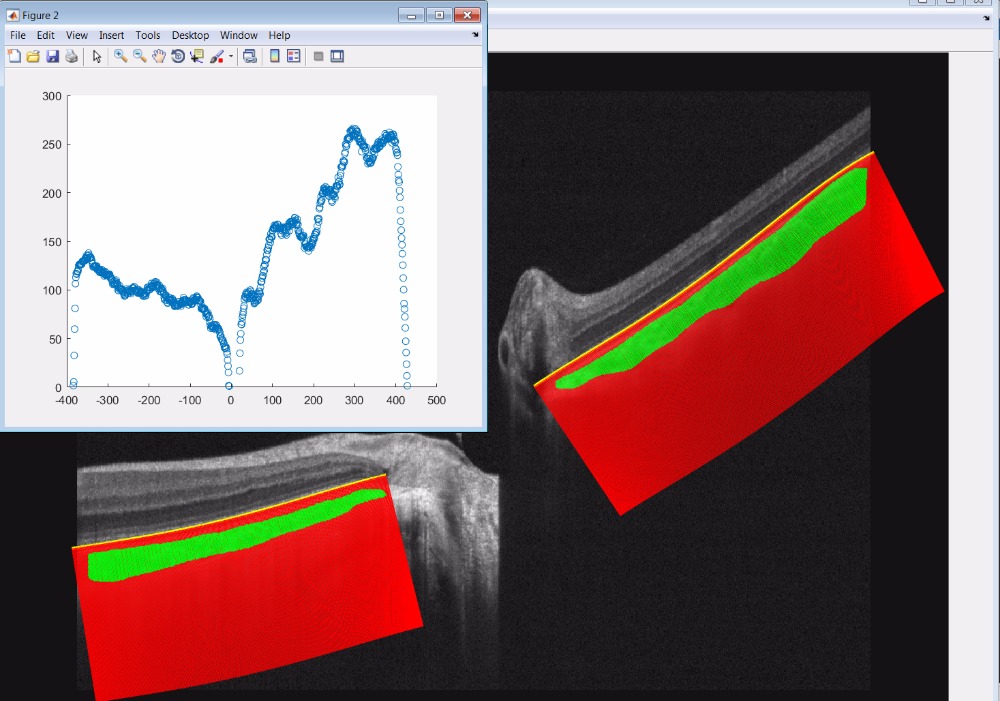


- 7 After running the script for all four cross lines for both eyes. Run ***OCT_Data_Comparison.m*** to generate interpolated data and compare restuls between two eyes. Choroid fhickness data in eight corners will be plotted and an interpolated picture will be shown. An xls file summarizing statistics will also be generated.


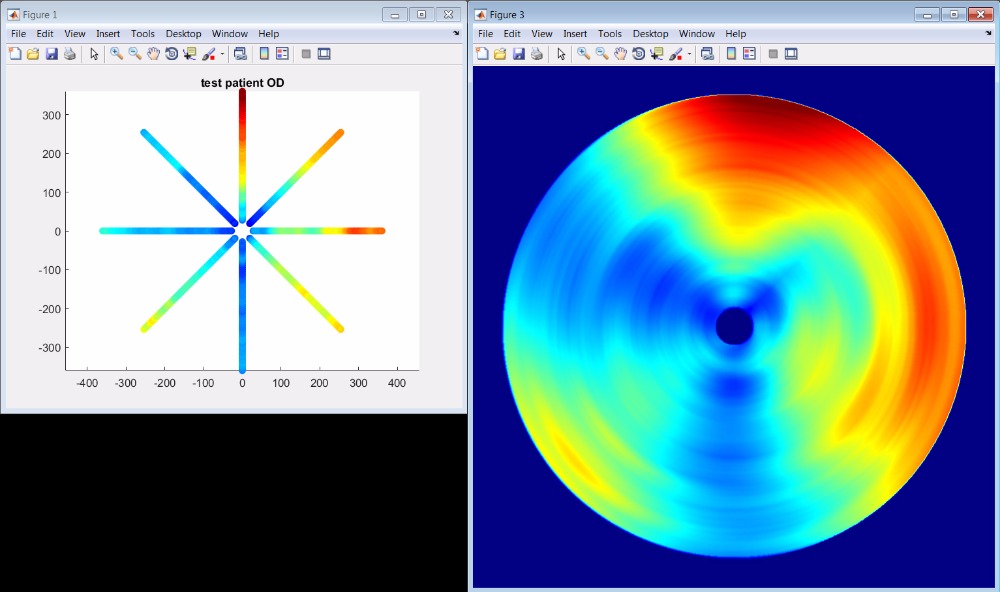

Supplement: S1 File — (DOCX) [file pone.0184927.s001.docx]
